# Supplementary material for: Nutritional supplement containing a nuclear fraction of bovine thymus gland increases the circulating levels of spermidine
Source: PLoS One. 2025 Sep 9;20(9):e0331813. doi: 10.1371/journal.pone.0331813 (PMC12419604; doi:10.1371/journal.pone.0331813)
Supplement: S1 Table — (DOCX) [file pone.0331813.s001.docx]

**S1 Table. Protocol for measuring encapsulated PAs in nutritional supplements using HPLC.**

| **1. Total free PA content**  (including insoluble precipitate and soluble content) | One gram of supplement powder was dissolved in perchloric acid, and the extracts were analyzed by HPLC for all 3 of the biogenic amines - putrescine, spermidine and spermine |
| --- | --- |
| **2. Total PA content**  (insoluble precipitate removed and liposomes destroyed) | A. One gram of supplement powder was dissolved in water at 100 mg/mL (10 mL)  B. The solution was centrifuged to remove the insoluble precipitate, and the supernatant was mixed with 0.5% triton X-100  C. Solution was centrifuged at 17,000 g for 10 min. The supernatant was used to perform putrescine, spermidine and spermine analysis by HPLC |
| **3. Encapsulated PA content** | A. One gram of supplement powder was dissolved in water at 100 mg/mL (10 mL)  B. The solution was first centrifuged to remove the insoluble precipitate. The supernatant was centrifuged at 150,000 g for 2 hrs to collect the liposomal nanoparticles  C. The nanoparticles were resuspended in water and mixed with 0.5% triton X-100  D. The solution was centrifuged at 17,000 g for 10 min and the supernatant was used to measure putrescine, spermidine and spermine by HPLC |
