# Supplementary material for: Nutritional supplement containing a nuclear fraction of bovine thymus gland increases the circulating levels of spermidine
Source: PLoS One. 2025 Sep 9;20(9):e0331813. doi: 10.1371/journal.pone.0331813 (PMC12419604; doi:10.1371/journal.pone.0331813)
Supplement: S2 Table — (DOCX) [file pone.0331813.s002.docx]

**S2 Table. LC elution gradients.**

| **Time (min)** | **A%** | **B%** |
| --- | --- | --- |
| 0 | 90 | 10 |
| 3 | 90 | 10 |
| 4 | 50 | 50 |
| 6 | 20 | 80 |
| 7 | 20 | 80 |
| 7.1 | 90 | 10 |
| 8.5 | 90 | 10 |
