# Supplementary material for: Nutritional supplement containing a nuclear fraction of bovine thymus gland increases the circulating levels of spermidine
Source: PLoS One. 2025 Sep 9;20(9):e0331813. doi: 10.1371/journal.pone.0331813 (PMC12419604; doi:10.1371/journal.pone.0331813)
Supplement: S3 Table — (DOCX) [file pone.0331813.s003.docx]

**S3 Table. MS parameters.**

| **Polyamine** | **Q1** | **Q3** | **Regression equation** | **Linear range (ng/mL)** | **R2** |
| --- | --- | --- | --- | --- | --- |
| Putrescine | 89.1 | 72.1 | y = 52387x - 6758.5 | 1-20 | 0.9904 |
| Spermidine | 146.2 | 72.1 | y = 105090x - 343041 | 2-50 | 0.9939 |
| Spermine | 203.1 | 129.1 | y = 36527x - 592454 | 10-200 | 0.9934 |
