# Supplementary material for: Nutritional supplement containing a nuclear fraction of bovine thymus gland increases the circulating levels of spermidine
Source: PLoS One. 2025 Sep 9;20(9):e0331813. doi: 10.1371/journal.pone.0331813 (PMC12419604; doi:10.1371/journal.pone.0331813)
Supplement: S4 Table — (DOCX) [file pone.0331813.s004.docx]

**S4 Table. DNA concentrations in nutritional supplements.**

| **Supplement** | **DNA conc. (ng/mL)** |
| --- | --- |
| SRS | nd* |
| TCF | 6.23438 |
| TNF | nd* |
| *nd - not detected | |
