# Supplementary figures and images for: Nutritional supplement containing a nuclear fraction of bovine thymus gland increases the circulating levels of spermidine
Source: PLoS One. 2025 Sep 9;20(9):e0331813. doi: 10.1371/journal.pone.0331813 (PMC12419604; doi:10.1371/journal.pone.0331813)

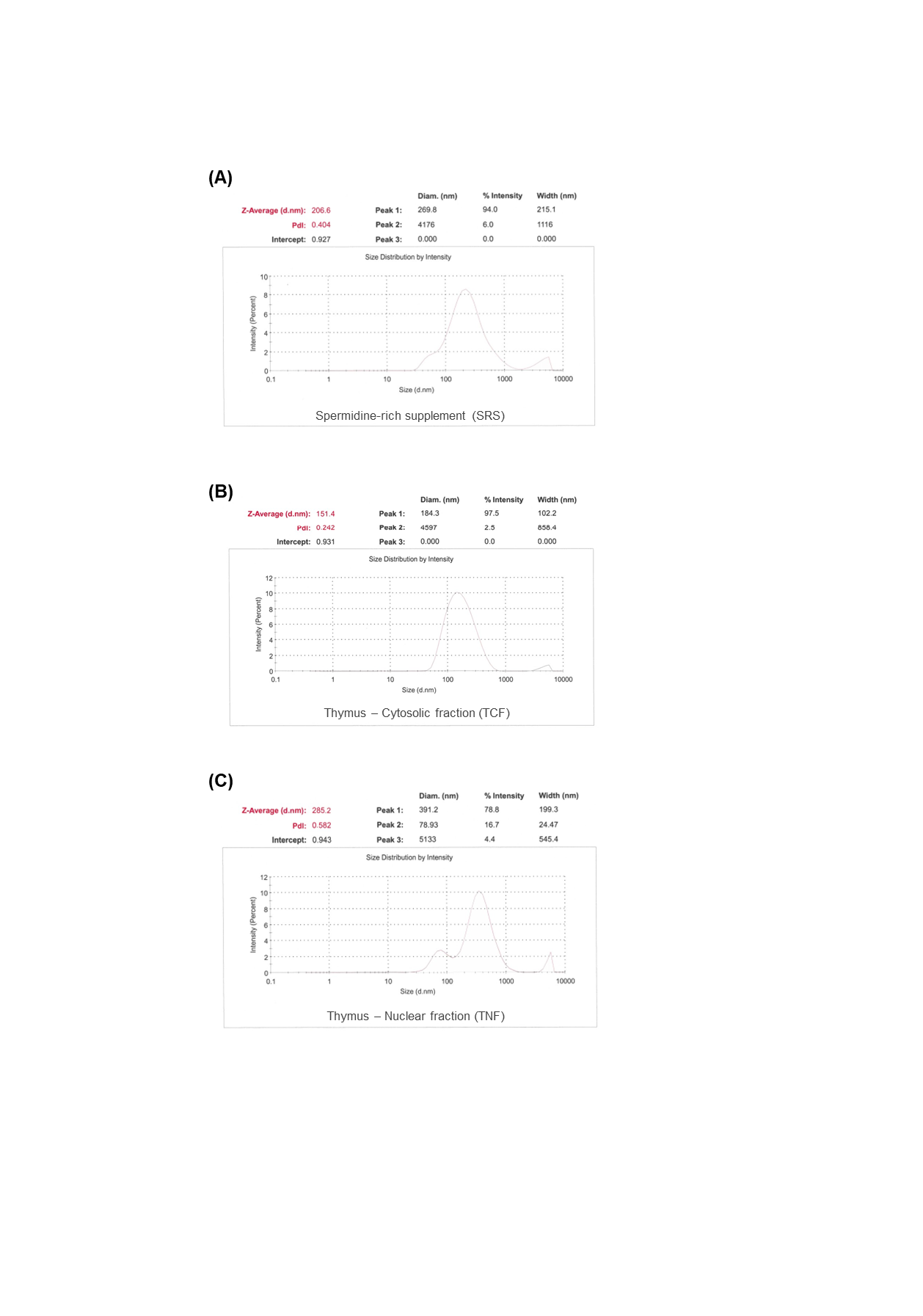

Supplement: S1 Fig — The dimensions of liposomes and their polydispersity indices (PDI) are shown for the SRS (A), TCF (B) and TNF (C). (TIF) [file pone.0331813.s005.tif]

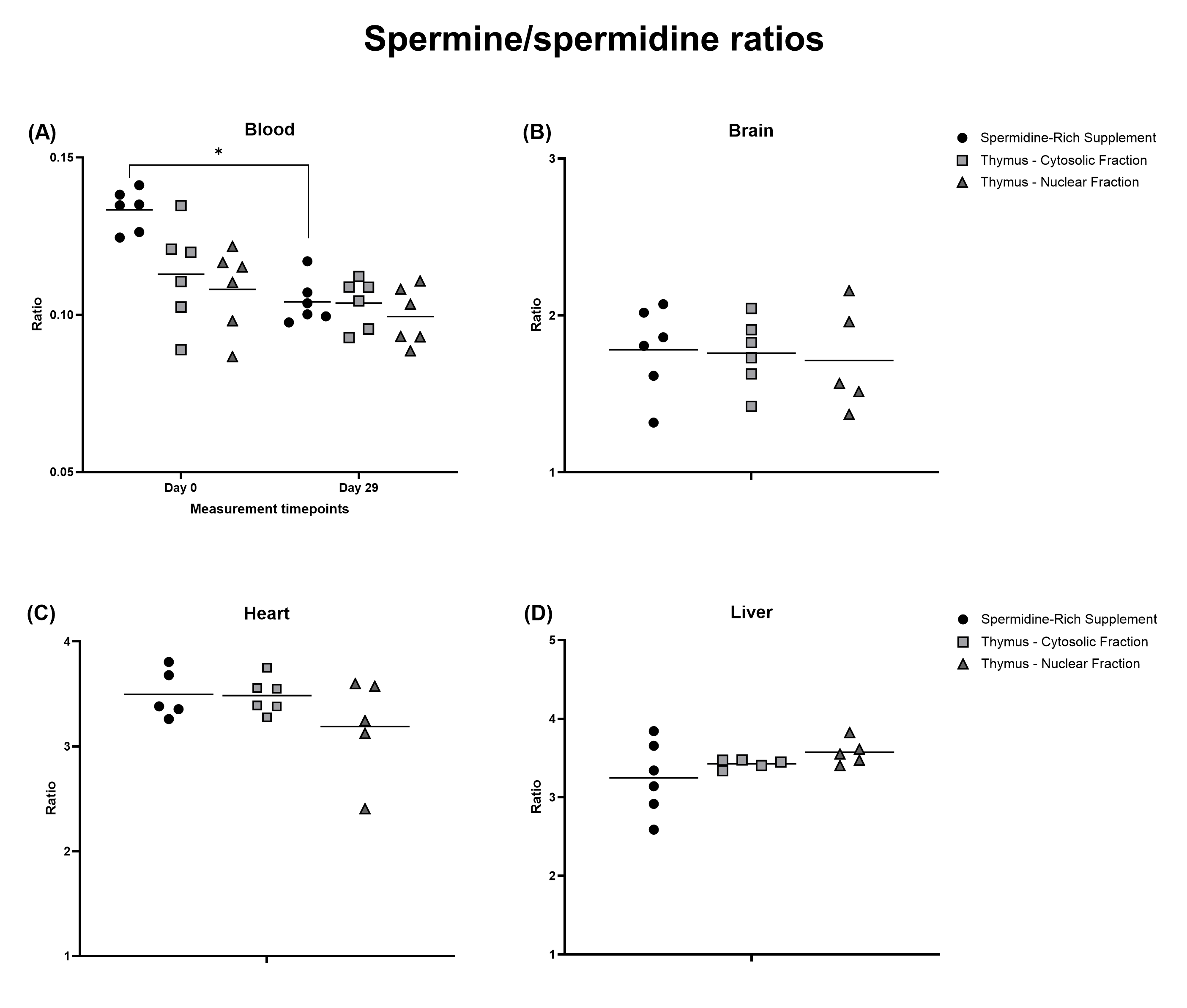

Supplement: S2 Fig — (A) Blood spermine/spermidine ratio was significantly reduced in SRS-supplemented animals compared to baseline, while no differences in spermine/spermidine ratios among the supplementation groups were detected in tissues: brain (B), heart (C) and liver (D). Data are shown as individual values and the mean; N = 4–6 animal per group. * - p < 0.0001 for effect of Time variable by Bonferroni/Dunn post-hoc analysis. (TIF) [file pone.0331813.s006.tif]
